# The role of gaming for information, education and communication of AMR: full review of online education resources

**DOI:** 10.1093/jacamr/dlae080

**Published:** 2024-06-11

**Authors:** Romita Trehan, Raphael Goujet, Tina Sharma, Abhinav Vats, Nidhiben Patel, Anshu Bhardwaj

**Affiliations:** Bioinformatics Centre, CSIR-Institute of Microbial Technology, Chandigarh 160036, India; Department of Life Sciences, Imperial College London, South Kensington, London, UK; Inserm, System Engineering and Evolution Dynamics, Université Paris Cité, Paris, France; Learning Planet Institute, Paris, France; Bioinformatics Centre, CSIR-Institute of Microbial Technology, Chandigarh 160036, India; Academy of Scientific and Innovative Research (AcSIR), Ghaziabad 201002, India; School of Biological Sciences, UM-DAE Centre for Excellence in Basic Sciences, University of Mumbai, Kalina Campus, Mumbai, Maharashtra, India; Inserm, System Engineering and Evolution Dynamics, Université Paris Cité, Paris, France; Learning Planet Institute, Paris, France; Bioinformatics Centre, CSIR-Institute of Microbial Technology, Chandigarh 160036, India; Inserm, System Engineering and Evolution Dynamics, Université Paris Cité, Paris, France; Learning Planet Institute, Paris, France; Academy of Scientific and Innovative Research (AcSIR), Ghaziabad 201002, India

## Abstract

**Background:**

The first objective of the Global Action Plan on antimicrobial resistance (AMR) is to improve awareness and understanding of AMR through effective communication, education and training. Towards this several efforts have been made to create AMR awareness resources. The aim of these resources is to inform the public about responsible antibiotic use and drive positive behavioural change. Digital media and specifically games can serve as unique innovative platforms in public communication programmes.

**Objectives:**

This study focuses on compiling and evaluating game-based AMR resources. Recognizing the engaging and creative potential of games as learning tools, the primary objective of this study was to identify games that can be used, individually or in combination depending on their unique focus and gameplay experience, for AMR awareness. Furthermore, games are evaluated on five objective criteria and recommendations are made towards further development of gaming resources towards AMR awareness.

**Methods:**

Meticulous curation was performed to mine information, education and communication resources, with a primary focus on games for AMR awareness and evaluating them based on game design and gameplay, AMR content and learning, engagement and replay appeal, learning outcomes, and level of difficulty and challenges.

**Results:**

In this study, we selected 12 AMR games. Our evaluations, spanning various gamification elements and interactive parameters, informed recommendations for future AMR resource development, including multilevel game design, varied graphics, simple-to-understand rules, sustained challenge and a sense of reward, among others.

**Conclusions:**

This study generated the first-ever comprehensive catalogue of AMR games that may assist public communication programmes for AMR awareness. Evaluation of these games led to actionable design recommendations for future resources towards effective communication of AMR complexity, enhanced learning and awareness.

## Introduction

Antimicrobial resistance (AMR) occurs when bacteria, viruses, fungi and parasites no longer respond to antimicrobial medicines.^1^ One of the primary reasons for AMR is the misuse and overuse of antibiotics, for example, poor adherence to the treatment guidelines and inadequate dosing.^2^ Other factors include self-prescription and misusing over-the-counter antibiotics, which are prevalent in low- and middle-income countries.^3^ In response to the global crisis of AMR, the World Health Assembly (WHA) proposed a Global Action Plan (GAP-AMR) in 2015 to be implemented under five major objectives, including: (i) efforts to increase awareness of AMR; (ii) enhanced surveillance and research; (iii) effective implementation of public health measures; (iv) responsible use of antibiotics; and (v) increase in investment towards better diagnostics, vaccines and therapeutics. As can be seen, one of the most important aspects of GAP-AMR is increasing awareness among all stakeholders to ensure concerted efforts in addressing this global scourge.

The age of antibiotics received global attention with the Nobel Prize-winning discovery of penicillin by Sir Alexander Fleming. This groundbreaking achievement ushered in a golden age of antibiotic development, witnessing the rapid introduction of numerous new antibiotics for human use. During this short period from the 1950s to 1970s, most of the antibiotics were discovered and introduced into clinical settings.^4^ Since then, concerns about AMR and its consequences have increased and it has now escalated into a global crisis. AMR being an evolutionary phenomenon, it is not surprising that bacteria adapted to and evolved several mechanisms to evade antibiotics.^5–7^ AMR has been a consistent focus for intervention since the 1990s, according to a recent assessment based on over 248 AMR policy documents and expert consultation reports. Since then, various major healthcare organizations have been introducing policy frameworks and legislation to bring attention to the issue of AMR. However, 81.5% of the reports are targeted towards governments, 5.2% towards the industry, and only 4.03% are aimed towards awareness among the general population, followed by healthcare, farmers/agriculture and academic sectors among others.^8^

The 2016 review on AMR, which was chaired by the economist Jim O'Neill, was one of the most referred reports on AMR. The report estimates that if antimicrobial drug resistance is not addressed, there will be 10 million deaths due to AMR by the year 2050 and an economic loss of between US$60 and US$100 trillion.^9^ Although these reports have served as wake-up calls not only for governments but also for the people directly involved in healthcare, by quantifying the economic and healthcare threats of AMR, they may not resonate as effectively with the general public. Hence there is a need for simpler, more comprehensive narratives that effectively communicate the gravity of AMR to the broader population. As reflected in the first objective of GAP-AMR, it is important to focus on raising awareness on AMR through public communication programmes that target different audiences in human health, animal health and agricultural practices as well as consumers, using effective information, education, and communication (IEC) strategies.

Over the years, there have been several efforts to create resources like posters and leaflets by WHO and other healthcare organizations promoting AMR awareness.^10,11^ These resources include both web-based and print media, like videos (YouTube animations explaining AMR),^12^ tutorials,^13^ infographics,^14,15^ comic books (like ‘The Antibiotic Tales) and pamphlets,^16^ program-like ‘Fun Kids Live’, massive open online courses (MOOCs) on antimicrobial stewardship,^17^ and websites with AMR learning resources^18^ and an Antimicrobial Resistance e-Learning Repository.^19^ In addition, gamification of concepts of how antibiotics work and how AMR emerges have also been done for enhanced engagement in understanding AMR.^20^ Gamification can be used to reinforce desired behaviours through relevant elements in a game (personalization, Proteus effect, etc.).^21^ It has been demonstrated that creative methods of engagement through games have the potential to simplify complex scientific concepts and have also been shown to increase awareness for AMR.^22^ Games have the potential to convey several concepts like ‘how bacteria infect people’ or ‘how AMR is spreading among the general public’, etc. Given that web-based media are gaining unprecedented popularity among the general public, this study focuses on the curation of web-based resources with a focus on AMR games. Subsequently, these games were evaluated based on various parameters like game design, gameplay, game elements, accessibility, replay appeal, AMR content and engagement, highlighting key elements from existing AMR games that suggest their use in different public communication programmes and also design principles for creating more engaging future resources.

## Methods

### Data source and curation

**Figure 1.**
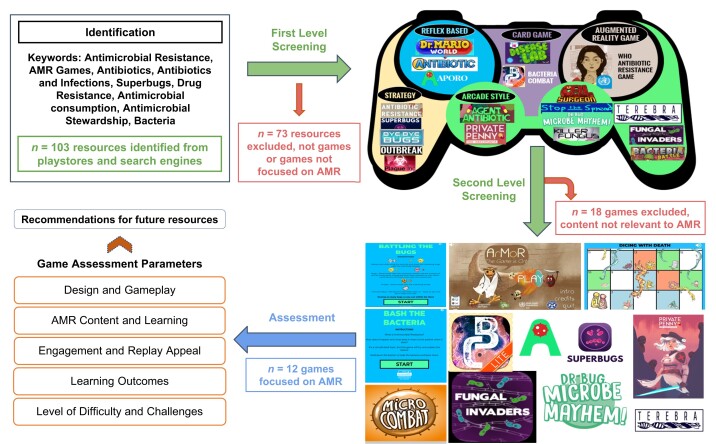
Flowchart depicting the workflow of the systematic curation of game-based resources on AMR awareness. Two-stage screening process is involved in selecting AMR games, which were then evaluated on several game parameters. The evaluation is then assessed to make recommendations for future resources of AMR.

In this study, we conducted a two-step curation process, illustrated in Figure 1. First a comprehensive list of AMR awareness resources spanning different multimedia formats was curated based on keywords searches—‘Antimicrobial Resistance’, ‘AMR Games’, ‘Antibiotics’, ‘Antibiotics and Infections’, ‘Superbugs’, ‘Drug resistance’, ‘Antimicrobial consumption’, ‘Antimicrobial, Stewardship’, ‘bacteria’ across several platforms including Google Play Store, iTunes and Google search (Supplementary material and Figure S1, available as Supplementary data at *JAC-AMR* Online). The curated data were stored in a structured manner including the name of the resource, category, genre, graphic style, country, dependencies (operating system) required, and the year and date of launch (Supplementary material and Figure S2). In total, the resources were curated with over 23 features including the name of the resource, their access details, graphic style, levels, update frequency, etc. (Supplementary material). In the second stage of the curation process, games were curated and analysed. Each game was reviewed by at least two authors. Objective evaluation criteria were then followed to evaluate games including the need for prior knowledge to play the game, graphics, replay appeal, gameplay, and learning components directly including concepts of AMR among others (Supplementary material). Although the gameplay experience may vary for each player, the collective assessment was presented as fun, boring or intermediate for each game. Popular gaming mechanisms like simple gameplay with low content complexity, clear and discrete interface, varying graphic styles, and a directed tutorial can enhance engagement and replayability. These aspects are crucial when developing games for AMR awareness to ensure player engagement.

### Game selection and analysis

The data curation primarily focused on video games with a focus on AMR awareness and education. Of the 30 games initially obtained through keyword search, the selection process further prioritized games focusing on AMR. This curation led to 12 games selected as tools for raising awareness and fostering understanding of AMR, forming the basis for further evaluation based on gameplay mechanics, replay appeal and learning outcomes. In addition, educational objectives, accessibility, difficulty level and the effectiveness of integrating AMR concepts into the gaming experience were also evaluated.

## Results

### Summary of the resources

A total of 103 resources with 12 games focusing on AMR were curated (Figure 1). The game resources were assessed on various parameters as mentioned in the Methods section. Most games are targeted towards children of various age groups.

Engaging children early on can help them develop a greater understanding and awareness of these issues. Studies have shown that children have a significant impact on how their parents, families and communities behave (intergenerational learning), clearly indicating that resources aimed at children are crucial for fostering community awareness.^23^ Thus, our study focused on video games as potential tools for blended learning. The 12 curated games offer a variety of gameplays and learning outcomes and are discussed below.

### Game assessment

**Figure 2.**
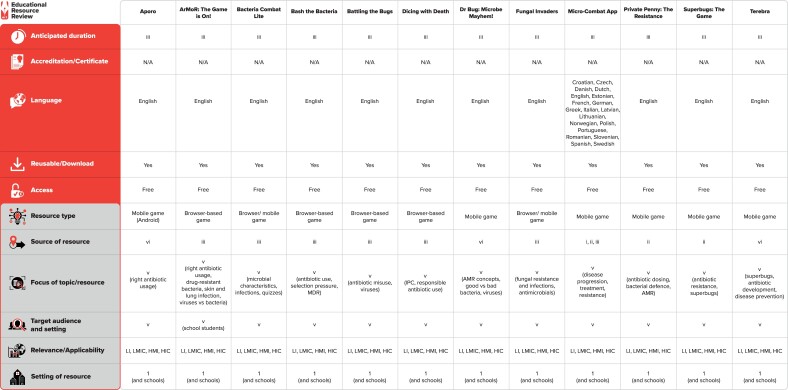
Summary of the resources reviewed. Resource web links: Aporo: https://play.google.com/store/apps/details?id=com.gioelecasazza.Aporo&hl=en&gl=US, ArMoR: The Game is On!: https://sites.google.com/view/anshub/armor, Bacteria Combat Lite: https://play.google.com/store/apps/details?id=com.GameDr.bacteriacombat&hl=en_IN&gl=, Bash the Bacteria: https://bugsgames.github.io/bashthebacteria/, Battling the Bugs: https://bugsgames.github.io/battlingthebugs/, Dicing with Death: https://bugsgames.github.io/dicingwithdeath/, Dr Bug: Microbe Mayhem!: https://portfolio.global-initiative.com/oxford-pharmagenesis  https://apps.apple.com/us/app/dr-bug-microbe-mayhem/id1248291732, Fungal Invaders: https://www.sciencegamecenter.org/games/fungal-invaders, Micro-Combat: https://eu-jamrai.eu/micro-combat/, Private Penny: The Resistance: https://play.google.com/store/apps/details?id=com.Ateneo.PPTR&hl=en_IN&gl=US, Superbugs: The Game: https://amr.longitudeprize.org/superbugs-game/, Terebra: https://play.google.com/store/apps/details?id=com.biofaction.synpeptide&hl=en&gl=US. **Source of resource:** (i) governments, (ii) professional societies, (iii) universities/higher education institutes, (iv) healthcare facilities, (v) WHO, (vi) industry, (vii) insurance companies, (viii) NGOs. **Focus of resource:** (i) principles/practice of prudent prescribing, (ii) antimicrobial stewardship principles/practices, (iii) guidelines/policies/pathways for syndrome management of infections, (iv) infection prevention/control, (v) implementation/behaviour change, (vi) evaluation/measurement, (vii) evidence gathering. **Target audience:** (i) doctors, (ii) pharmacists, (iii) nurses/midwives, (iv) non-medical managers, (v) public health, (vii) laboratory, (viii) infection prevention practitioners. **Setting of resource:** 1. Pre-service (university, higher education institution), 2. Service (i) hospital, (ii) outpatient clinic, (iii) community/general practice, (iv) long-term care facility/nursing home, (v) hospital and ambulatory, (vii) other. HIC, high-income countries; HMI, high- and middle-income countries; IPC, infection prevention and control; LI, low-income countries; LMIC, low- and middle-income countries; N/A, not applicable.

The effectiveness or impact of a game on learning or awareness relies on several factors. Therefore, assessment of all 12 games was done and discussed in the context of game design and game play, AMR content and learning objectives, engagement and replay appeal, learning outcomes and level of difficulty. In addition, a summary of the resources reviewed is presented in Figure 2.

#### Design and gameplay

Well-designed core mechanics are crucial for creating games with compelling gameplay.^24^ When discussing the game design of these educational/awareness games focused on AMR it is essential to evaluate their gameplay and interactive elements. These games revolve around the theme of battling microbes and incentivizing responsible antimicrobial usage with higher scores or levels. ‘Fungal Invaders’ offers simple shooting mechanics to eliminate fungi whereas ‘Superbugs: The Game’ has a simple game design that involves a screen tapping method to kill bacteria that appear in the Petri dish displayed on the screen, with more resistant bugs appearing over time. ‘Dr Bug: Microbe Mayhem!’ displays a magnified view of the gut to eliminate harmful microbes using antibiotics or WBCs and also highlights the importance of healthy food towards fighting infections. ‘Private Penny: The Resistance’ is a 2D survival shooter game where an antibiotic protagonist defends against waves of increasingly resilient bacteria using upgradable antibiotics. ‘Bacteria Combat Lite’ engages players in card battles featuring bacteria, with opportunities to regain lives through questions and special cards. ‘Micro-Combat App’ is a multiplayer card game that challenges players to strategize disease progression by countering bacteria attacks with defence cards in a time-bound manner. ‘Bash the Bacteria’ is a multilevel game with each level introducing the impact of antibiotics on the emergence of resistant bacteria. The player is given the choice of selecting antibiotics to treat resistant and susceptible forms by double-clicking to eliminate bacteria. ‘Dicing with Death’ is a dice-based game (like snakes and ladders) where the player navigates through several scenarios highlighting that positive behaviour, reflected through appropriate antibiotic use, is a winning strategy in the game. ‘Battling the Bugs’ penalizes the players for targeting unidentified bugs with random antibiotics and rewards them for avoiding antibiotic misuse. ‘Aporo’ involves colour-matching antibiotics to bacteria to prevent the creation of more challenging superbugs. Super antibiotics are also provided but the player is cautioned against their frequent use. ‘Terebra’ is a jigsaw puzzle-based game with a core gameplay mechanism that involves creating peptide antibiotics by assembling amino acids. Players must link these amino acids in a specific sequence and fold them to form effective ‘molecular crowbars’ capable of destroying bacteria. One of the latest in the repertoire of AMR games is ArMoR, a multilevel game in a tower defence game design context, requiring players to strategically deploy antibiotics to combat pathogenic organisms. The game offers two terrains (tissue types) so far, skin and lung. ArMoR has varied graphics and different game elements across levels to introduce the complex phenomenon of AMR in a progressive manner. Each game offers a unique gameplay conveying the message of responsible antibiotic use. These games have a lot of potential to promote AMR awareness through easy access and easy-to-understand instructions.

#### AMR content and learning

Each game incorporates educational elements related to AMR to raise awareness about the issue. In ‘Fungal Invaders’ and ‘Superbugs: The Game’, players witness the evolution of stronger fungi and bacteria, respectively, due to continuous antimicrobial use, emphasizing the selective pressure that drives AMR. ‘Dr Bug: Microbe Mayhem!’ illustrates the impact of antibiotics on gut bacteria, demonstrating that antibiotic overuse harms good bacteria, promotes superbugs and emphasizes healthy food choices. ‘Private Penny: The Resistance’ underscores the importance of taking the correct antibiotic dose to prevent bacterial resistance and highlights the significance of responsible antibiotic usage and research. ‘Bacteria Combat Lite’ provides players with information on various bacteria through card descriptions and quizzes, fostering knowledge about bacterial characteristics. ‘Micro-Combat App’ highlights the challenge of treating drug-resistant infections. ‘Bash the Bacteria’ demonstrates how selection pressure from antibiotics can lead to MDR bacteria. ‘Dicing with Death’ conveys the importance of infection prevention and control and responsible antibiotic use. ‘Battling the Bugs’ demonstrates that antibiotics work only on bacteria, and that their misuse leads to harder-to-treat future infections. In ‘Aporo’, players learn the importance of using the correct antibiotics by matching colours to avoid creating difficult-to-treat bugs, reinforcing responsible antibiotic use. The puzzle-based game mechanics of ‘Terebra’ are used to convey the complexity in designing new antibiotics and the threat of AMR in a global context. ArMoR offers comprehensive AMR-related content, covering various aspects of bacterial invasion, division, emergence of AMR and resistance mechanisms. It effectively communicates the significance of responsible antibiotic use. Thus, these games collectively offer engaging ways to educate players about AMR and its implications and effectively communicate the importance of responsible antibiotic use.

#### Engagement and replay appeal

Several factors contribute to the effectiveness of AMR games as AMR/AMS resources, most importantly, the overall player experience.^24^ Engaging gameplay that effectively integrates AMR concepts offers the best chance of achieving learning outcomes and raising awareness. ‘Fungal Invaders’ and ‘Superbugs: The Game’ employ strategy and reflex-based techniques, with escalating difficulty levels that keep players engaged and challenged. ‘Dr Bug: Microbe Mayhem!’ offers fast-paced gameplay with clear AMR concepts, ensuring high player engagement and replay value. ‘Private Penny: The Resistance’ combines smooth and modern graphics with interactive elements like survival, shooter, upgrade mechanics and resource management maintaining player interest despite progressively difficult gameplay. ‘Bacteria Combat Lite’ provides knowledge about bacteria but can become overwhelming, potentially decreasing player interest. It may be suited for a classroom setting where the purpose is to educate about specific infections and pathogenic organisms. ‘Micro-Combat App’ engages players through stages of disease progression and treatment cards under time pressure and multiplayer modes making learning about AMR competitive. ‘Bash the Bacteria’, being a multilevel time-bound game, has a strong replay appeal and is engaging and informative for players with a good balance of simplicity and challenge. ‘Dicing with Death’ is a two-player game that offers a unique gameplay where the random outcomes of rolling dice lead to engagement and strong replay value. ‘Battling the Bugs’ provides engaging gameplay with increasing difficulty and reinforces the importance of using the right antibiotic. ‘Aporo’ combines challenge and time-pressure to keep players engaged in the importance of antibiotic selection. ‘Terebra’ keeps players engaged with challenging gameplay, urgency, strategic decision-making, and the opportunity to compete for high scores, ensuring good replay appeal. ‘ArMoR’, being a multilevel game, excels in engagement, immersing players in an exciting gaming experience that also imparts valuable knowledge about antibiotics, resistance and related topics, ultimately turning out to have high replay appeal. However, Aporo and Dicing with Death were comparatively boring. Overall, these games offer a mix of engaging experiences and educational content, with varying degrees of replay appeal.

#### Learning outcomes

All games evaluated in this study offer valuable learning outcomes related to AMR and antibiotic use. In ‘Fungal Invaders’ the players learn about fungal resistance and the consequences of antimicrobial misuse, highlighting the need for new treatments as resistance emerges. ‘Superbugs: The Game’ demonstrates how the rise of superbugs is inevitable and the overuse and misuse of antibiotics are fast-forwarding their evolution. ‘Dr Bug: Microbe Mayhem!’ teaches players about antibiotics, superbugs and the importance of healthy food in combating bad bacteria. ‘Private Penny: The Resistance’ reinforces the concept of taking the right antibiotic dose to prevent resistance and the importance of sustained research into AMR. ‘Bacteria Combat Lite’ provides basic information about microbes, infections and their effects. ‘Micro-Combat App’ imparts knowledge about the consequences of antibiotic misuse and the importance of timely and correct treatment. ‘Bash the Bacteria’ teaches players about antibiotic-resistant bacteria and the selection pressure of antibiotic use. ‘Dicing with Death’ demonstrates the importance of several aspects of antibiotic use as well as responsible behaviour like infection prevention and control. ‘Battling the Bugs’ educates players on antibiotic misuse and that antibiotics do not work on viruses. ‘Aporo’ emphasizes the importance of using the right antibiotics to avoid the development of drug-resistant bugs, fostering an understanding of responsible antibiotic use. In ‘Terebra’ the players are made aware of the impending threat of AMR and the complexity of designing new antibiotics. ‘ArMoR’ aims to educate players about bacterial infection, and the importance of using antibiotics in a timely and appropriate manner. Being a multilevel game, each level is designed to introduce a new scientific concept related to antibiotics, bacterial infections and resistance, ensuring that players grasp these crucial ideas in a stepwise manner while enjoying the game play. This game also has a dedicated level to convey that antibiotics do not act on viruses. Depending on their learning outcomes, these games can be effectively used to meet specific AMR-related educational objectives in lesson plans and tailor communication strategies, reinforcing the key messages from the games and enhancing public understanding of scientific concepts and AMR impact.

#### Level of difficulty and challenges

The level of difficulty takes into account the ease of understanding gameplay based on the instructions provided as well as the game interface, which varies across these games. ‘Fungal Invaders’ offers a moderate challenge as players deal with progressively stronger fungi, although controlling the syringes can be tricky. ‘Superbugs: The Game’ is relatively easy to understand but presents some challenges as bacteria become more resistant. ‘Dr Bug: Microbe Mayhem!’ combines simplicity with moderate difficulty, demanding quick decision-making. ‘Private Penny: The Resistance’ is a moderately challenging game with fast-paced and competitive game mechanics to destroy increasingly resistant bacteria. ‘Bacteria Combat Lite’ ranges from moderate to difficult due to information overload and quiz-based life regeneration. ‘Micro-Combat App’ presents a moderate challenge as players prevent infection spread. ‘Bash the Bacteria’ strikes a balance between easy and moderate difficulty, ensuring an engaging experience. ‘Dicing with Death’ leans towards the easier side given that it is based on a popular game format. ‘Battling the Bugs’ offers a moderate challenge as players match antibiotics to different bacteria. ‘Aporo’ also falls into the moderate category, emphasizing the importance of proper antibiotic use through colour matching. In ‘Terebra’ players are expected to know about antibiotics, AMR and why new antibiotics are needed. The gameplay progressively becomes harder. ‘ArMoR’ strikes a balance with a moderate level of difficulty, ensuring that players can grasp the educational content without feeling overwhelmed. Instructive elements in the game are important for player engagement and a positive gameplay experience.^25^ Whereas these assessments are based on descriptions, actual effectiveness would require playtesting and comparisons based on objective scoring mechanisms like SUS.^26^

## Discussion

Misuse and overuse of antibiotics have emerged as a major reason for the spread of AMR especially affecting the middle- and low-income countries. As per the recommendations of the GAP-AMR, awareness resources are critical to align policy interventions with participation from the public. Towards this, online resources have emerged as a potential tool for awareness, and therefore in this study the most engaging mode of awareness, i.e. games, were curated and evaluated. Our curation led to a comprehensive list of 12 AMR games. Based on evaluation of various parameters, the following recommendations are suggested for further development of AMR games. The games should be multilevel, to easily introduce complex AMR phenomena gradually over the course of the game. This approach would also increase the duration of gameplay, which along with smooth graphics and consistent gameplay increases the replay appeal of a game and keeps the user engaged. This should also be complemented by simple-to-understand and integrated rules that are easier to follow. Finally, informative learning games should be designed so that minimal prior knowledge is required for a positive game onboarding experience. In addition, the player should get a sense of achievement and therefore rewards (e.g. scores and leaderboards); these are also critical to engaging players, with each game displayed in terms of points and level progression. Engagement is key to learning and therefore a strong replay appeal is at the core of the learning experience and is critical to convey the complex phenomenon of AMR and how this issue impacts everyone, in ways that are not well understood by the general public, leading to a progressive crisis. To improve the player experience, the overwhelming challenges or obstacles in the game can be balanced by providing occasional moments of respite or rewards to manage the adrenaline levels and maintain engagement.

Games serve not only as valuable resources for researchers studying the effectiveness of gamification in AMR education but also as dynamic tools for teachers to captivate students and effectively convey complex concepts. For scientific teams focused on public awareness, these games offer a powerful bridge between scientific knowledge and public understanding of AMR to even younger audiences. Their potential to encourage judicious antibiotic use makes them valuable tools in the fight against AMR. Integrating these gamified resources into public awareness campaigns and interactive events may further amplify community engagement. The versatility of these resources extends beyond research settings, finding applicability in workshops, classrooms and various digital learning platforms. As such, these gamified tools emerge as invaluable assets for researchers, educators, science communicators and initiatives dedicated to public engagement, thereby significantly contributing to heightened awareness and enhanced understanding of AMR. Therefore, in this context, this study offers a list of curated resources on AMR awareness that may assist public communication programmes towards increasing AMR awareness.^27,28^ As internet accessibility through mobile phones is increasing, the adoption of mobile games is on the rise, which positions games to be effective tools to reach an even wider audience. For this to be feasible, games are preferred to be accessible over low-bandwidth connections with platform-independent technologies. The advent of Open-AI (artificial intelligence) platforms can further revolutionize and democratize development of new games and other immersive modes of engagement.^29^

It is important to highlight that games are not just tools for awareness but can also be used for data collection. They can be used to streamline the practices of medical professionals, as recently recommended,^30^ with real-time monitoring of antibiotic prescriptions, diagnostic assessments, patient follow-ups, etc. In addition, it is important that evidence-based antibiotic prescription is followed, which requires precision diagnosis for both existing and new resistant infections. As already discussed, the Global Action Plan has awareness as one of its core pillars. However, it is imperative to highlight that other pillars of the GAP-AMR are also critical to addressing the complex issue of AMR under the One Health umbrella.

## Supplementary Material

dlae080_Supplementary_Data
