# Supplementary material for: The role of gaming for information, education and communication of AMR: full review of online education resources
Source: JAC Antimicrob Resist. 2024 Jun 11;6(3):dlae080. doi: 10.1093/jacamr/dlae080 (PMC11165311; doi:10.1093/jacamr/dlae080)
Supplement: dlae080_Supplementary_Data [file dlae080_supplementary_data.zip › Supplementary_File_S1_CLEAN.docx]

**Distribution and Curation Summary**

**
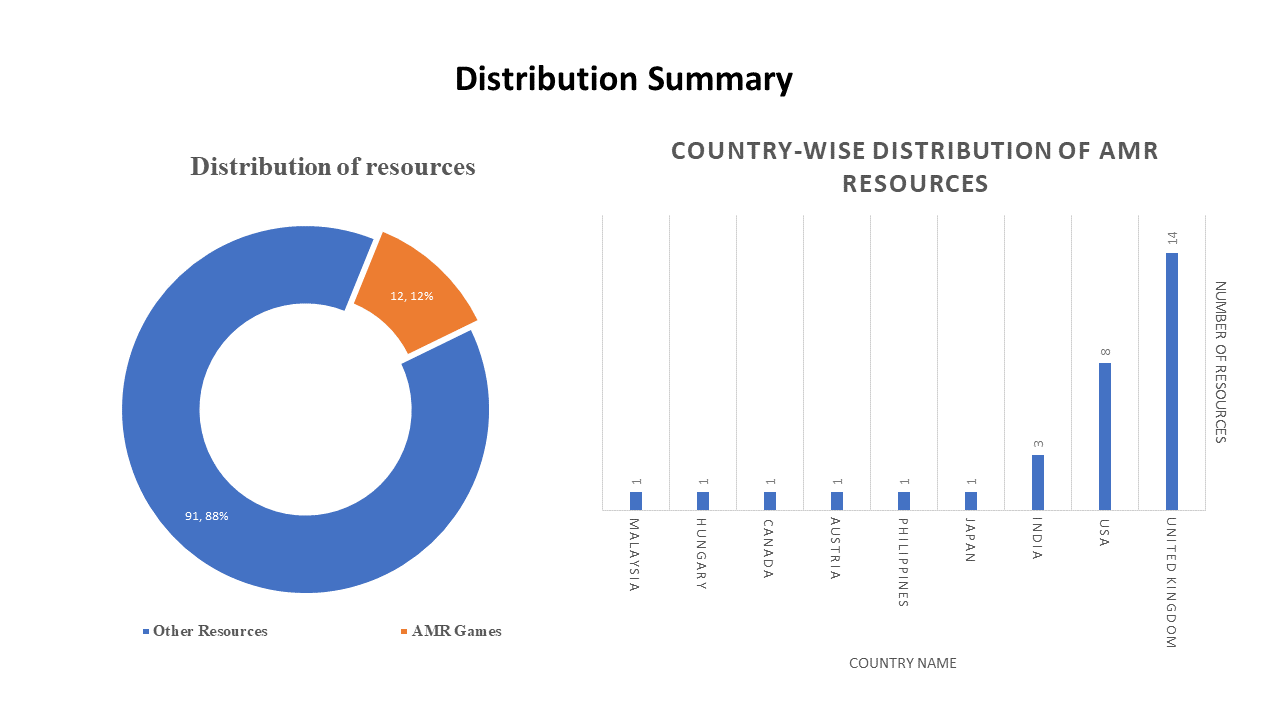
**

**Supplementary Figure 1**: Distribution of 103 resources on Antimicrobial Resistance awareness available over the internet. These resources were collected from across the internet through keyword searches. Country of origin was available for only 31 out of our list of 103 resources. Of these, 12 games focus on AMR awareness. The rest of the curated resources include resources like the Antimicrobial Guide by Stanford for dosing information on infection types and allergies, ^1^ videos like WHO’s Science in 5 on AMR released during the COVID-19 pandemic, ^2,3^ timely released as the attention to AMR had already started declining around 2017-2018. ^4^ Animated videos like “How can we solve the antibiotic resistance crisis” by Gerry Wright ^5^  and “The antibiotic apocalypse explained” by Kurzgesagt- In a Nutshell ^6^ simplify AMR. These resources also emphasise that antibiotic use in animals, not just humans, contributes to drug resistance, even affecting last-line antibiotics like colistin. ^7^


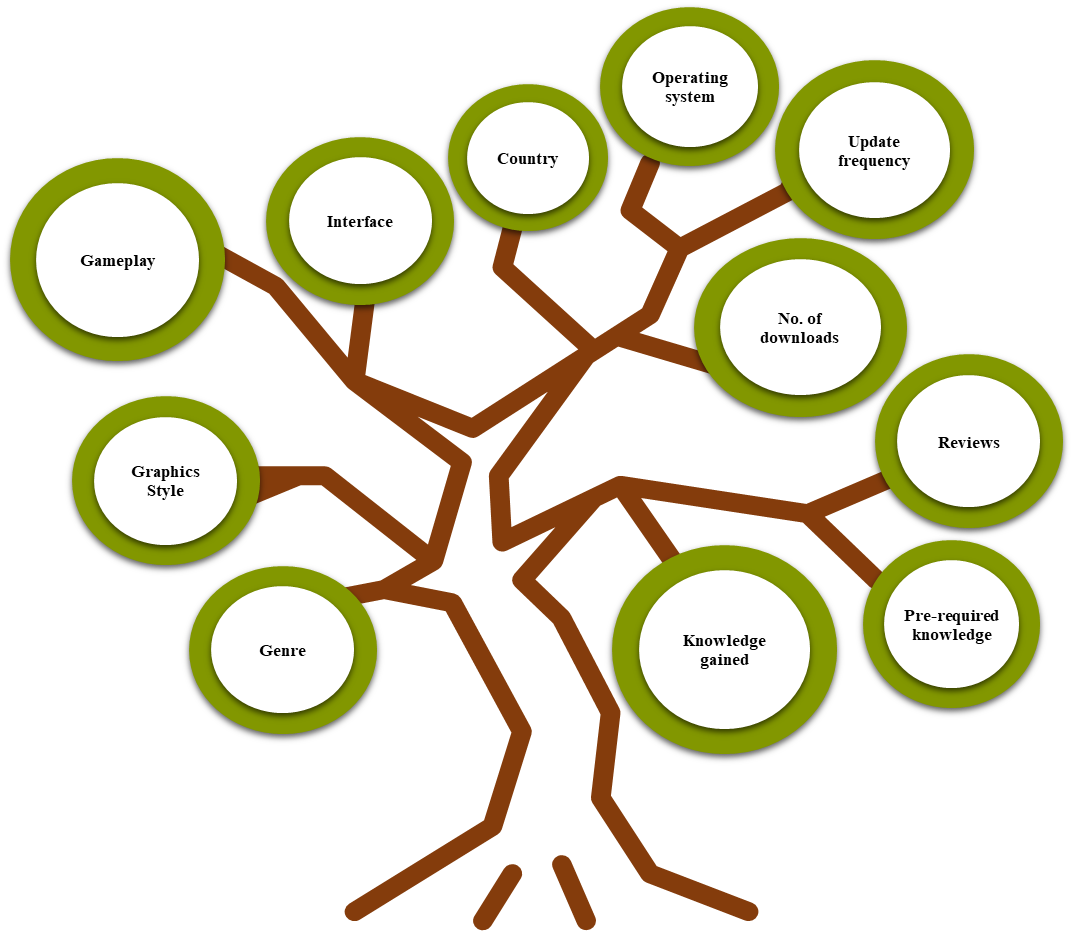


**Supplementary Figure 2:** Few Parameters of evaluation of AMR resources.

**References**

1. Stanford Antimicrobial Safety & Sustainability Program SM. Antimicrobial Guidebook. Available at: https://med.stanford.edu/bugsanddrugs/guidebook.html.

2. World Health Organization (WHO). WHO’s Science in 5 : COVID-19 & Antibiotics - YouTube. 2021. https://www.youtube.com/watch?v=R3Q5ETOm33M.

3. World Health Organization (WHO). WHO’s Science in 5 on COVID-19 - Antibiotics & COVID-19- YouTube. *World Health Organization* 2020. https://www.youtube.com/watch?v=lFpLIDQcdyQ.

4. Overton K, Fortané N, Broom A, *et al.* Waves of attention: Patterns and themes of international antimicrobial resistance reports, 1945-2020. *BMJ Glob Health* 2021; **6**.

5. Gerry Wright, Artrake Studio, Artrake Studio T-E. How can we solve the antibiotic resistance crisis?- YouTube. 2020. https://www.youtube.com/watch?v=ZvhFeGEDFC8.

6. Kursgesagt- In a Nutshell. The Antibiotic Apocalypse Explained - YouTube. *2016* 2016. https://www.youtube.com/watch?v=xZbcwi7SfZE.

7. Dawadi P, Bista S, Bista S. Prevalence of Colistin-Resistant Escherichia coli from Poultry in South Asian Developing Countries. *Vet Med Int* 2021; **2021**.
